# Unsheltered Homelessness and Health: A Literature Review

**DOI:** 10.1016/j.focus.2022.100043

**Published:** 2022-10-29

**Authors:** Jessica Richards, Randall Kuhn

**Affiliations:** Department of Community Health Sciences, Jonathan and Karin Fielding School of Public Health, University of California Los Angeles, Los Angeles, California

**Keywords:** Homelessness, unsheltered homelessness, health, social determinants of health

## Abstract

**Context:**

In recent years, cities across the world have seen widespread growth in unsheltered homelessness, in which a person sleeps in a place not meant for human habitation such as cars, parks, sidewalks, and abandoned buildings. It is widely understood that people experiencing homelessness have poorer health outcomes than the general population. Less is known about the health of people who are unsheltered, yet they may be exposed to greater health risks than their sheltered counterparts. The purpose of this literature review is to evaluate and summarize the evidence on unsheltered homelessness and health.

**Evidence Acquisition:**

A literature search was conducted using PubMed to identify publications on unsheltered homelessness and health. A total of 42 studies were included for review.

**Evidence Synthesis:**

Unsheltered populations experience higher rates of chronic disease, serious mental illness, and substance abuse than sheltered populations. Unsheltered homelessness is strongly associated with chronic homelessness that exacerbates serious mental illness and substance use, which is often co-occurring. Despite having large unmet health needs, unsheltered populations have lower healthcare utilization and often lack health insurance.

**Conclusions:**

Evaluating the impact of shelter status on health outcomes has important implications for the allocation of housing and health services. Longitudinal studies are needed to examine the relationship between the duration of sheltered and unsheltered homelessness and health outcomes and explore the mediating mechanisms that lead to poor health among unsheltered populations. Despite these limitations, our results also suggest an urgent need to address the unique and severe challenges facing unsheltered populations and the need for intervention approaches that are sensitive to these unique disease burdens.

## HIGHLIGHTS

• Unsheltered homelessness is rising, particularly in the Western U.S.
• We reviewed studies that compared health outcomes by shelter status.
• Unsheltered populations have higher rates of physical and mental illness and substance use.
• Unsheltered populations use health care less and often lack health insurance.
• Longitudinal studies are needed to assess the causes of poor health among unsheltered persons.

## CONTEXT

In recent years, cities across the world have seen widespread growth in unsheltered homelessness.^1^ Above and beyond the epidemiologic risks associated with homelessness itself,^2^, ^3^, ^4^ unsheltered individuals may experience additional disease burdens relating to exposures such as violence, exploitation, weather, pollution, and poor sanitation. Yet, few studies have established the health consequences of unsheltered homelessness, much less their extent or underlying mechanisms.^5^ This literature review evaluates and summarizes the small but growing body of literature on health outcomes among unsheltered homeless adults, specifically in comparison with those who are sheltered.

**Figure 1:**
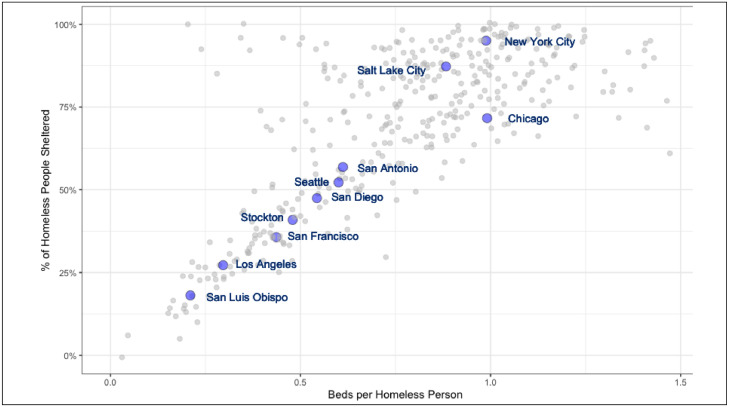
Relationship of homeless shelter bed inventory with unsheltered homelessness, U.S. Continuums of Care, 2020.

Source: U.S. Department of Housing and Urban Development. Point-in-time count and housing inventory Count Data; 2020. *HUD Exchange*. https://www.hudexchange.info/programs/hdx/pit-hic/.

The past decade has seen a sizable increase in the proportion of the U.S. homeless population who are unsheltered. The U.S. Department of Housing and Urban Development defines an individual as homeless if they lack a fixed, regular, and adequate nighttime residence.^1^ Within this category, those who sleep in a public or private place not meant for human habitation (e.g., street, tent, or other makeshift shelters) are considered unsheltered. According to the U.S. Department of Housing and Urban Development's Annual Homeless Assessment Report, the unsheltered homeless population increased by 30% from 2015 to 2020, even as the sheltered homeless population declined by 10%. Therefore, the share of unsheltered persons nationwide rose from 31% to 39%. Aggregate data mask wide geographic variations in the distribution of unsheltered homelessness, with higher rates in the Western U.S. Although it is widely assumed that unsheltered homelessness results from warmer weather, Figure 1 suggests a simpler relationship whereby localities with more shelter beds will have a lower share of their homeless population unsheltered.^6^ Indeed, New York City had much higher rates of unsheltered homelessness in the 1990s, which were reduced because of a concerted effort to build shelters and engage clients.^7^ Recent increases in unsheltered homelessness partially track a series of rulings by the U.S. 9th Circuit Court of Appeals, which struck down urban camping prohibitions until shelter beds were made available to house the entire homeless population.^8^

Countries define and measure homelessness differently, making cross-country comparisons of unsheltered homelessness difficult. The definition of homelessness varies by country, and identification of the unsheltered homeless population is beset by limited data sources and methodologic challenges.^9^ In some countries, the definition of homelessness may include only people who are unsheltered (e.g., Japan) and unsheltered and sheltered populations (e.g., U.S.) or be applied broadly to also include precariously housed populations (e.g., Australia). Countries with a more inclusive definition of homelessness tend to report a higher incidence of homelessness.^10^ Although unsheltered persons are commonly included in official definitions of homelessness, they are often not separately identifiable in national indicator data outside of the U.S.^11^ Unsheltered homelessness has been referred to as street homelessness,^12^ absolutely homeless,^13^ rooflessness, sleeping rough,^14^ or long grassing,^15^ and in turn, individuals have been referred to as rough sleepers, street/pavement dwellers, and encampment residents. Many individuals may also live in both sheltered and unsheltered locations at different points in time or even at the same time.^16^ These differences in operationalizing homelessness will be considered when drawing comparisons across studies.

Where possible, we focus on studies that disentangle the impacts of unsheltered homelessness from confounding factors associated with unshelteredness. Unsheltered persons are more likely to be non-Hispanic White, male, and veteran than those who are sheltered and have a history of incarceration or foster care.^5^^,^^17^, ^18^, ^19^, ^20^ Duration may also serve as a confounder because unsheltered homelessness is associated with prolonged and more frequent episodes of homelessness.^5^^,^^17^^,^^18^^,^^20^

This review pays particularly close attention to chronic health conditions affecting older adults. Recent studies have framed the long-term consequences of homelessness in terms of accelerated aging because of repeated exposure to deprivation and disease, as reflected in the early onset of geriatric conditions and surgical complication risks often occurring decades sooner than in housed older adults.^21^^,^^22^ The population of older homeless adults (aged ≥50 years) is also growing in the U.S. owing to ongoing cohort effects.^23^

## EVIDENCE ACQUISITION

We organized this literature review following Fazel and colleagues’ 2014 review of health outcomes for the broader homeless population.^2^ Results were grouped into the following health outcomes: mortality, noncommunicable diseases, reproductive health, communicable disease, mental health, substance abuse, health services utilization, and injuries.

### Search Strategy

**Figure 2:**
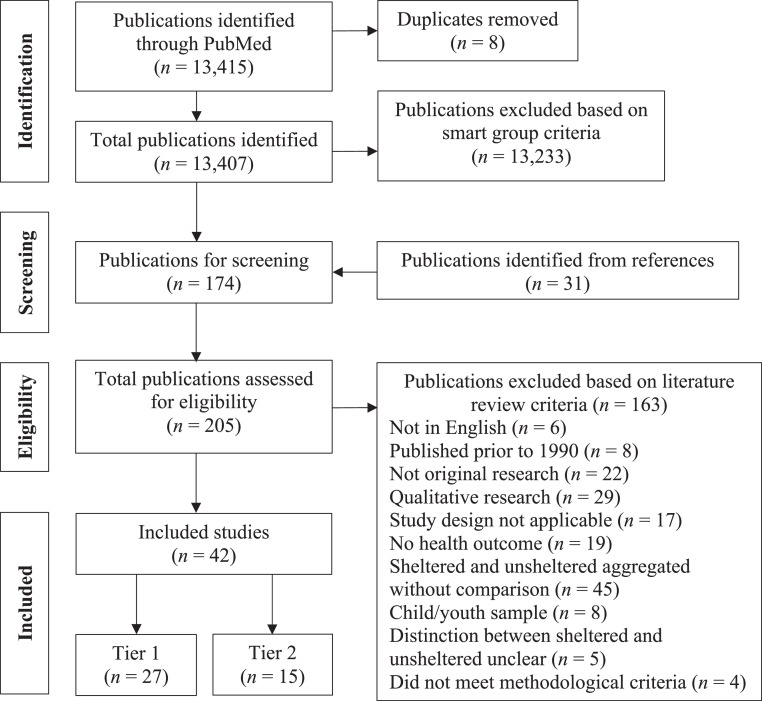
Summary of search strategy and study selection.

The literature search strategy and study selection are summarized in Figure 2. A literature search was conducted in May 2020 using PubMed to identify publications on unsheltered homelessness from 1990 through 2020. Combinations of relevant keywords including unshelter* and rough sleeper* and street homeless* were used to capture variations of unsheltered homelessness. A total of 13,415 publications were identified. Keywords for each search are listed in Appendix Table 1 (available online). Grouping, deduplication, and coding were conducted in EndNote to minimize the risk of errors or lost data. The reviewer (JR) consulted with an experienced biomedical staff librarian to translate PubMed searches into EndNote smart groups (Appendix Table 2, available online). Publications on unsheltered homelessness were identified as the intersection of publications identified using keywords for homelessness and publications identified using keywords for unshelteredness (Appendix Table 2, available online). A total of 174 publications were identified for screening to identify studies in which a health or health utilization measure was the outcome of interest. To address the potential risk of publication bias, we conducted parallel searches of the gray literature using Google Scholar and found no additional studies.

### Study Selection

The full text for all the 174 publications was located and read to determine eligibility for review. Reference lists were also searched to identify relevant publications and an additional 31 publications were screened for review. Following earlier studies, the review included only studies that estimated an association between unsheltered homelessness and a health-related outcome.^24^ Studies that did not clearly meet the inclusion criteria were discussed with a second reviewer (RK) and any disagreements were resolved by consensus. For transparency, the list of 132 excluded studies is included in Appendix Table 3 (available online), along with the criteria for exclusion.

### Study Coding

Included studies were grouped into 2 tiers: (1) comparative studies of unsheltered and sheltered homelessness and (2) studies with an exclusively unsheltered sample. Tier 2 evidence was only reported if it contradicted Tier 1 evidence or if Tier 1 evidence did not exist for a health domain.

Owing to a wide range of methodologic rigor within the included studies, a scoring system was developed to evaluate study quality within each tier. Specifically, papers were scored on (1) the rigor of the sampling strategy, (2) the use of validated health measures, and (3) efforts to control for or otherwise account for (e.g., through standardization) the role of population composition. Initially, sampling rigor was coded using probability sampling of a known population. However, few studies met this requirement. Thus, the standard was relaxed to code studies on the basis of the following hierarchy: (1) probability sampling or quota sampling occurred at a mix of known venues, and/or efforts were taken to compare the resulting sample with more representative samples of the population of interest (e.g., point-in-time homeless counts); (2) convenience samples where the sample was not selected on the basis of health or health risk (i.e., certain neighborhoods of a city); and (3) convenience samples where the sample was selected on the basis of health risk (i.e., substance abuse program). Studies were independently scored by both reviewers (JR and RK), and discrepancies were resolved by consensus. Studies with a score of 1 were excluded from analysis. The inclusion criteria and scoring are listed in Appendix Table 4 (available online). In all, 42 publications were selected for review (Tier 1: 27, Tier 2: 15).

## EVIDENCE SYNTHESIS

The review includes 42 studies. Thirteen of them were Tier 1 comparative studies with quasi-representative sample design, 14 were Tier 1 comparative studies with convenience samples, and 15 were Tier 2 studies with unsheltered samples only (Supplementary Electronic Appendix, available online). Results for Tier 1 studies are reported using adjusted (if available) odds/risk ratios for the unsheltered versus sheltered comparison. For Tier 2 studies, we report unadjusted estimates for the unsheltered population.

**Figure 3:**
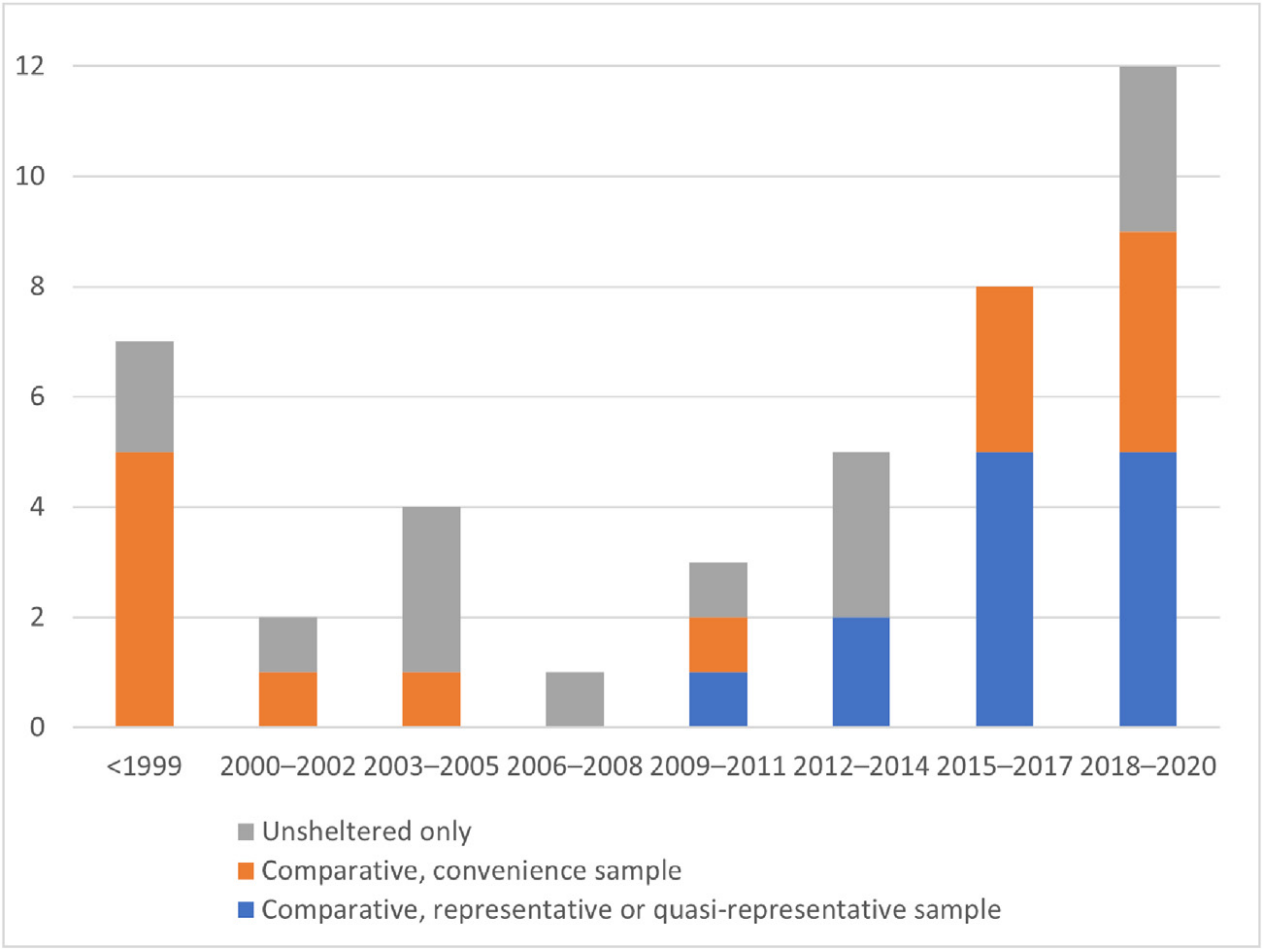
Included studies by year and quality.

Figure 3 shows that more than half of the studies, including all Tier 1 representative studies, were conducted in the past decade. Nearly half of the studies (20 of 42) were published since 2016, including 10 of the 13 comparative quasi-representative studies. Nearly three quarters of the studies took place in the U.S. (29 of 42), including 85% of studies published in the past 5 years (17 of 20) and 85% of comparative quasi-representative studies (11 of 13).

**Figure 4:**
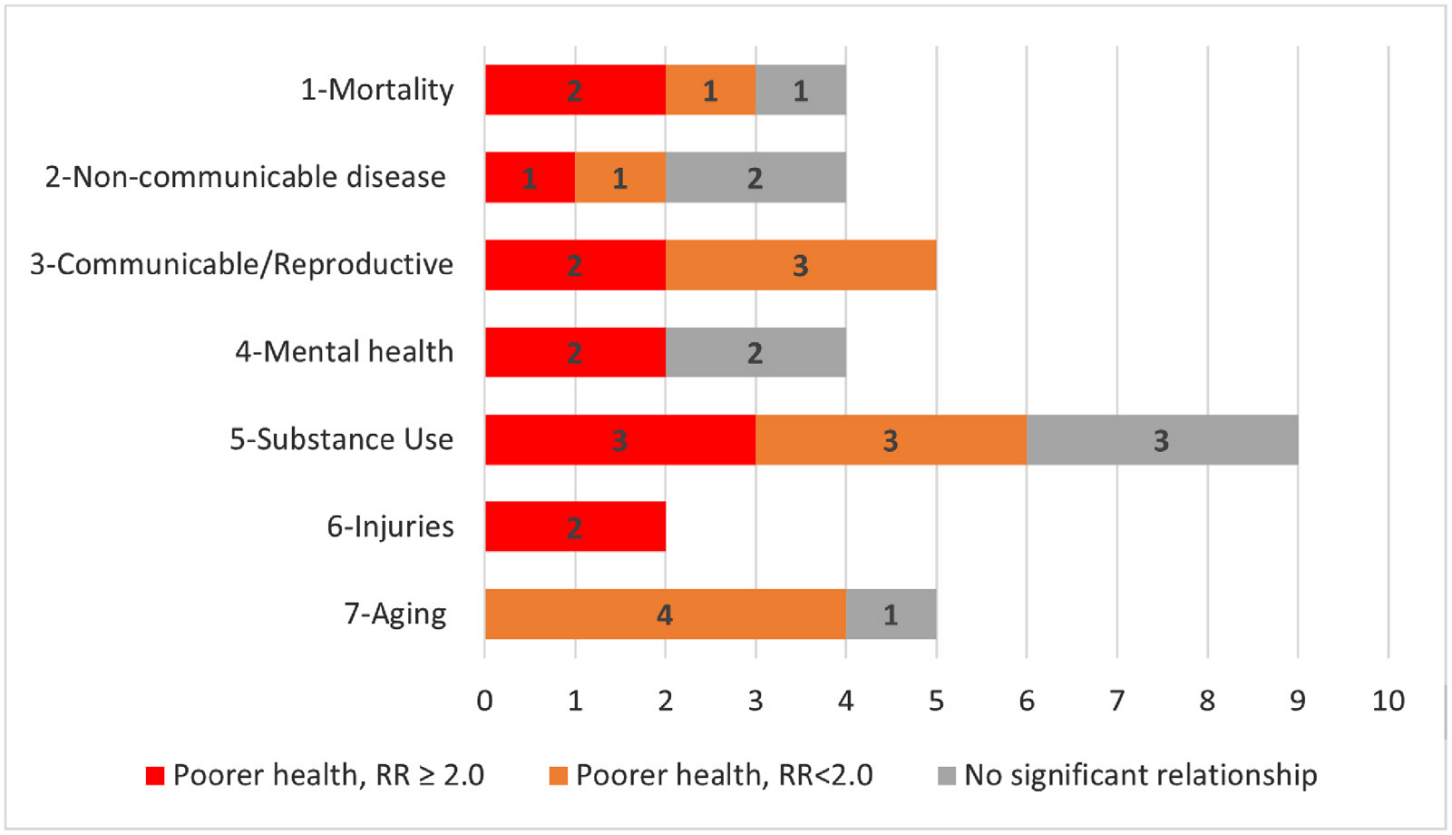
Classification of single associations for each health outcome by type of relationship support and outcome of interest.

We extracted 48 unique directional associations from the 27 Tier 1 comparative studies we reviewed (Figure 4). We summarized the type of relationship supported for each finding for all health outcomes. We classified results as those supporting substantially poorer health for unsheltered (statistically significant with RR/OR≥2.0), somewhat poorer health for unsheltered (significant with RR/OR between 1.0 and 2.0), and not statistically significant. No Tier 1 studies provided support for unsheltered individuals having better health than sheltered comparators. Across all the 48 Tier 1 findings, 44% (21 of 48) found that those who are unsheltered had much poorer health, 29% (14 of 48) supported moderately but significantly poorer health, and 27% (13 of 48) found no significant relationship. Within each of the 8 health outcome groups, most findings indicated significantly worse health for unsheltered than sheltered, although the number and quality of findings varied by outcome.

### Mortality and Cause of Death

Mortality rates are significantly higher among those experiencing unsheltered homelessness. Compared with that of a sheltered homeless cohort, the standardized mortality ratio (SMR) for an unsheltered homeless cohort in Boston was nearly 3 (SMR=2.7; 95% CI=2.3, 3.2) times higher.^25^ After grouping the unsheltered sample by mortality risk factors, the SMR for unsheltered versus that for sheltered was 4.0 (95% CI=3.0, 5.2) times higher for a high-risk group and 2.2 (95% CI=1.8, 2.8) times higher for the lower-risk cohort.^26^ A national study using data from the 100,000 Homes campaign found a significant but much smaller effect of unsheltered status on the odds of mortality (AOR=1.12; 95% CI=1.05, 1.19).^17^

Three papers addressed the cause of death.^25^, ^26^, ^27^ Common causes of death among those who were unsheltered included chronic disease, substance use, and injuries.^25^ Compared with nonpoisoning injuries among a sheltered cohort, nonpoisoning injuries (e.g., motor vehicle accidents, falls, drowning) were high (SMR=7.1; 95% CI=4.4, 11.0) and were associated with high rates of chronic substance use (SMR=4.2; 95% CI=2.5, 6.7).^25^ Unsheltered adults classified as high risk had substantially higher mortality rates for HIV/AIDS (SMR=122.3; 95% CI=44.8, 271.1), chronic substance use (SMR=104.2; 95% CI=38.1, 231.0) primarily caused by alcohol abuse, chronic liver disease (SMR=86.0; 95% CI=45.0, 150.0), and injuries (SMR=44.0; 95% CI=17.8, 91.6) than a sheltered high-risk cohort.^26^

### Noncommunicable Diseases and Associated Markers

Unsheltered populations often experience poor adult health outcomes. A cross-sectional study of homeless women in Los Angeles found that after controlling for sociodemographic factors (e.g., age, education, ethnicity, number of times homeless, and length of time homeless), unsheltered women had greater odds of fair or poor physical health (AOR=3.40; 95% CI=2.34, 4.94; *p*=0.001) and experiencing pain in the last 6 months (AOR=2.28; 95% CI=1.54, 3.37; *p*=0.001) than sheltered homeless women.^28^ The association between unsheltered status and worse health status remained significant for women who used substances (AOR=3.0; 95% CI=2.02, 4.45) and women with poor mental health (AOR=2.24; 95% CI=1.49, 3.37).^28^ Another study conducted in Wales found that oral health‒related quality of life was significantly poorer among rough sleepers than among sheltered homeless adults (*p*=0.004).^29^ Other common self-reported medical problems among the unsheltered include orthopedic problems and arthritis.^30^

Evidence is mixed regarding the impact of shelter status and chronic homelessness on chronic health conditions.^17^^,^^31^^,^^32^ A study of chronically homeless veterans found that sheltered veterans were more likely to have a chronic health condition than unsheltered (43.4% vs. 40.8%; *p*=0.001), although this result was not controlled for confounders.^19^ Although a comparative study of chronically unsheltered and not chronically unsheltered adults did not find significant differences in the rates of serious medical conditions, chronically unsheltered adults were significantly more likely to report trimorbidity (e.g., serious medical issue, lifetime mental illness, lifetime substance abuse) than not chronically unsheltered adults (OR=1.65; 95% CI=1.11, 2.45).^20^ A study of air pollution‒related health outcomes among people experiencing homelessness found that breathing difficulty and headaches did not vary significantly on the basis of shelter status (shelter versus unsheltered) or chronicity (chronic versus nonchronic homelessness).^33^

A number of studies focused on older homeless adults.^19^^,^^20^ One study found significantly higher rates of vision impairment among unsheltered older adults (*p*=0.04).^21^ However, *mobility impairment* (defined as self-reported difficulty in walking) and other geriatric conditions (activities of daily living impairment, instrumental activities of daily living impairment, one or more falls in the past 6 months, cognitive impairment, hearing impairment, urinary incontinence, or depression) did not vary significantly by living environment.^21^ Among unsheltered older adults, 34% reported falling once or more in the last 6 months,^21^ and spending any night unsheltered (compared with none) was significantly associated with increased odds of falling (AOR=1.42; 95% CI=1.10, 1.83).^34^ Unsheltered older adults had more than twice the odds of very low food security than older adults who were recently homeless or staying in temporary accommodation or institution.^35^

### Communicable Disease

Just 4 papers addressed communicable diseases, with just 2 Tier 1 studies and 2 conducted in the U.S. One paper addressed unsheltered‒sheltered differences in tuberculosis risk and treatment.^36^ People living on the street had the greatest risk of tuberculosis compared with those housed and other homeless groups (sheltered and transient), and the average number of days hospitalized and required for follow-up care was 4 times as high as that of sheltered homeless.^36^ Two descriptive studies conducted in Ethiopia observed that 44%–68% of street dwellers had 1 or more intestinal parasites.^37^^,^^38^

### Sexual and Reproductive Health

Although only 3 studies examined sexual and reproductive health, results suggest that some high-risk sexual behaviors and rates of sexually transmitted diseases may be more common, particularly for women. In the Los Angeles study, unsheltered women had greater odds of having multiple sex partners (AOR=2.79; 95% CI=1.93, 4.03; *p*=0.001) and having a sexually transmitted disease (AOR=2.10; 95% CI=1.05, 4.21; *p*=0.036) in the past 6 months.^28^ They were also more likely to have experienced an unwanted pregnancy (AOR=1.53; 95% CI=1.07, 2.19; *p*=0.021).^28^ Among unsheltered women experiencing reproductive health problems in India, most (78.5%) did not seek care.^39^ A descriptive study of street dwellers in Ethiopia indicated that 39.4% had experienced sexually transmitted disease symptoms in the past year.^40^

### Mental Health

Unsheltered homelessness is often accompanied by high rates of mental health illness, including major depression. In the Los Angeles study, unsheltered women had much greater odds of being in poor mental health (AOR=12.69; 95% CI=6.68, 24.13; *p*=0.001) than sheltered homeless women.^28^ A study of unsheltered adults in Japan found that street homelessness was significantly associated (OR=2.64; 95% CI=1.15, 6.06; *p*<0.05) with recent suicidal ideation after controlling for depression.^12^ In addition, in a descriptive study of unsheltered adults in Ethiopia, 41.8% wished to die, 21.7% had persistent suicidal thoughts, and 14.8% had attempted suicide in the past month,^14^ but another study found that the rates of lifetime major depression did not vary significantly between unsheltered and sheltered men.^41^ A study of older homeless adults found that the rates of depression and suicidal thoughts did not vary significantly between unsheltered and those in other living groups, but the study did not include a clearly sheltered comparison group.^21^

In addition to major depression, schizophrenia and mood disorders are common mental health diagnoses among unsheltered populations. High prevalence rates have been found in descriptive studies across 3 countries. Most Brazilian unsheltered adults had a psychiatric diagnosis (98.8%); an Ethiopian study found that 41.0% had psychosis; and among a small sample of rough sleepers in Dublin, 31.3% had a severe mental illness.^14^^,^^42^^,^^43^ Among those with mental illness, the most common diagnoses were schizophrenia (88% in Ethiopia, 25% in Dublin, and 9.6% in Brazil) and mood disorders, including major depression (32.5% in Brazil).^14^^,^^42^^,^^43^

Chronicity may contribute to the rates of mental illness among unsheltered persons. Exploratory analysis indicated that adults with psychosis in Ethiopia were more likely to be older and to have longer durations of street homelessness.^14^ Chronically unsheltered individuals were more likely to have *lifetime mental illness* (defined as either a history of psychiatric hospitalization or current mental health counseling or treatment) (OR=1.57; 95% CI=1.19, 2.08) than not chronically unsheltered persons.^20^ In contrast, a recent study found mental health illness did not vary by shelter status (shelter versus unsheltered) or chronicity (chronic versus nonchronic homelessness).^33^

### Substance Use

Experiencing unsheltered homelessness can significantly increase the likelihood of substance use disorder and worsen existing substance use. In 1 study, unsheltered women were more likely to use alcohol or noninjection drugs in the past 6 months (AOR=2.95; 95% CI=1.94, 4.50) than their sheltered counterparts, but no significant differences were found regarding recent injection drug use.^28^ Among opioid-using veterans in New York City, being unsheltered was a significant predictor (adjusted incidence rate ratio=2.08; 95% CI=1.39, 3.13) of greater engagement in opioid overdose risk behaviors after adjusting for demographics and prescription medications.^44^ A study of sheltered and unsheltered tobacco users found few differences in tobacco use in the previous month, with the exception that unsheltered smokers were significantly more likely to report using large cigars (AOR=2.35; 95% CI=1.05, 5.23).^45^ A study of young adults found that sheltered young adults were significantly less likely to have used alcohol (AOR=0.53; 95% CI=0.34, 0.82), marijuana (AOR=0.53; 95% CI=0.34, 0.83), and synthetic marijuana (AOR=0.46; 95% CI=0.25, 0.84) in the past month than unsheltered participants after controlling for other factors. The rates of stimulant and opioid use were also lower but were not statistically significant.^46^

Among a cohort of out-of-treatment substance users, a composite HIV risk score (number of times injecting drugs, number of days using crack, and number of days having sex) was significantly higher for all unsheltered subgroups (Black males, Hispanic males, White males, Black females, Hispanic females) except for White females (*p*<0.05) than for sheltered participants.^47^ Another national study of substance users found that unsheltered homelessness was strongly associated with frequent public drug use (AOR=17.44; 95% CI=9.5, 32.0) compared with stably housed participants after controlling for age and use of heroin or injection drugs.^48^

Comorbid mental health and substance use disorder is also common among unsheltered populations.^14^^,^^42^^,^^43^ Chronically unsheltered adults were more likely to have a dual diagnosis of mental illness and substance use (*p*=0.002) than not chronically unsheltered adults.^20^ In addition, veterans with a substance use disorder, alone or in combination with a mental illness, were significantly more likely to be unsheltered, although this did not apply to veterans with only a mental illness.^19^ The Los Angeles study found that unsheltered women with recent substance use had much greater odds (AOR=11.09; 95% CI=5.62, 21.88) of poor mental health than sheltered women with recent substance use.^28^

The most used substance among unsheltered populations is alcohol.^30^^,^^49^^,^^50^ The rates of alcohol use were high, with 68% of rough sleepers in London^49^ and 72% of encampment residents in Los Angeles^30^ reporting using alcohol in the past month. Other commonly used drugs among the unsheltered include crack cocaine, heroin, and cannabis.^30^^,^^49^^,^^50^ Drugs are often used in combination among unsheltered individuals, using an average of 3 or 4 drugs.^50^

There is evidence that substance use may increase with the duration of unsheltered homelessness. Among unsheltered adults in London, longer durations of unsheltered homelessness were accompanied by increased daily substance use, injection drug use, and dependency.^50^ Substance use was a commonly reported cause of homelessness, and 80% reported using at least 1 additional drug since homelessness onset.^49^

### Injuries

Little evidence documents levels of injury risk for unsheltered and sheltered individuals. In the Los Angeles study, unsheltered women were more likely to have experienced victimization in the form of physical assault (AOR=2.74; 95% CI=1.91, 3.94; *p*=0.001) and robbery (AOR=5.37; 95% CI=3.64, 7.92; *p*=0.001) than sheltered women.^28^ Although half of the unsheltered homeless adults in Manhattan reported a history of repeated trauma, the rates did not significantly vary between chronically unsheltered and not chronically unsheltered homeless adults.^20^

### Health Services

**Healthcare access and utilization.** Unsheltered homelessness has been associated with lower rates of healthcare utilization. Not residing on the streets was significantly associated with recent health services utilization (AOR=11.39; 95% CI=3.58, 36.24) after adjusting for socioeconomic factors and other covariates in a study based in South Korea.^51^ After adjusting for sociodemographic and homelessness characteristics, women experiencing unsheltered homelessness were less likely to have seen a dentist in the past year (AOR=0.34; 95% CI=0.21, 0.53; *p*=0.001) and to have received a Pap test (AOR=0.40; 95% CI=0.28, 0.59; *p*=0.001) or a tuberculosis test (AOR=0.22; 95% CI=0.15, 0.33; *p*=0.001) than their sheltered counterparts.^28^ After adjustment, unsheltered individuals in England were less likely to be registered with a general practitioner (AOR=0.45; 95% CI=0.30, 0.66) than sheltered participants but were not less likely to utilize primary care services.^52^ Unsheltered older adults had significantly lower rates of primary care services than older homeless adults in other residential categories.^53^ Yet, regarding follow-up care, unsheltered clinic users were more likely (1.45 times) to return for care than those staying in a sheltered environment after controlling for other factors.^54^

Findings for hospital-related health service use among unsheltered homeless individuals are mixed. Homeless veterans in Los Angeles with a history of sleeping unsheltered had lower odds of using inpatient services (OR=0.34; *p*=0.002) than sheltered veterans,^55^ whereas a bivariate analysis found no difference in the use of outpatient services among homeless veterans on the basis of shelter status.^19^ Another study found higher rates of outpatient services among an unsheltered cohort but less use of emergency services and fewer hospital admissions than among sheltered adults.^56^ One study found higher rates of health services utilization among the unsheltered, including emergency department and outpatient services, but this effect was primarily explained by high levels of chronic health conditions.^5^ A study of rough sleepers in England also found no association between shelter status and use of hospital care, hospital admissions, emergency services, or ambulance use after adjusting for covariates.^52^ Unsheltered populations are also less likely to have health insurance.^19^

Among current or past drug users, unsheltered women were less likely to have sought formal treatment in their lifetime than those sheltered (AOR=0.31; 95% CI=0.21, 0.47).^28^ In an unadjusted analysis, unsheltered homeless persons were significantly more likely to report not receiving needed substance use treatment than sheltered homeless persons (61.0% vs. 45.6%; *p*<0.001).^57^

## DISCUSSION

Despite the mixed quality of reviewed studies, our review suggests a consistent and strong association between unsheltered homelessness and higher levels of health risk, above and beyond the well-documented negative consequences of homelessness.^2^ Unsheltered populations experience high rates of chronic disease, serious mental illness, and substance abuse compared with sheltered populations. Despite having many unmet health needs, unsheltered populations have lower healthcare utilization and often lack health insurance. These health disadvantages manifest in significantly higher burdens of mortality.^26^ Unsheltered homelessness is strongly associated with chronic homelessness, which exacerbates serious mental illness and substance use, which are often co-occurring.

### Limitations and Future Studies

We note a number of concerns surrounding methodologic quality. First, few studies addressed the critical outcomes of injuries, communicable diseases, and sexual and reproductive health. Second, we observed substantial variation in the definition of unshelteredness and of comparison groups, so comparisons between sheltered and unsheltered populations should be interpreted with care. A variety of measures have been used to determine shelter status. Unsheltered homelessness has been based on current living situation,^58^ previous night location,^46^ and having slept within a certain area.^40^ Other studies incorporate duration data by gathering residential histories to identify where participants sleep most of the time. Residential time windows range from within the past 1 week,^45^ 1 month,^28^ 3 months,^57^ and even 6 months.^31^ Finally, we note the variable quality of the sampling methods. Few studies used a probability sample. Only half of the comparative studies reviewed employed methods for gaining quasi-representativeness, either by sampling through multiple venues or comparing the sample to point-in-time estimates of target population composition. Some recent studies with consistently higher-quality evidence have produced more mixed results.

Future studies must employ longitudinal designs to address causal mechanisms linking unsheltered homelessness to health through specific pathways of risk. The potential for reverse causality underscores the need for longitudinal studies to explore temporal relationships. During the pandemic, many jurisdictions have targeted vulnerable individuals for placement in shelters or hotels, potentially altering the temporal relationship between health and unshelteredness.^59^^,^^60^ Yet, at the same time, homeless services systems often impose rules or restrictions that cause vulnerable groups to avoid or be removed from shelters. This can include well-known restrictions such as sobriety requirements as well as more subtle barriers such as pet ownership restrictions that may exclude even those with service animals.^61^

An even more important need is to identify and address the specific social‒ecologic exposures that drive poor health among the unsheltered and how these exposures interact with shelter status. Only 1 paper in this review attempted to isolate a causal factor in the relationships between health and sheltered/unsheltered homelessness.^41^ Notably, risk factors such as chronic exposure to low-quality food and sleep disruption because of light and noise pollution have not been addressed at all. Future studies should leverage longitudinal data where possible and disaggregate people experiencing homelessness by shelter status to further explore the mechanisms that drive poor health among unsheltered populations. A better understanding of these mechanisms would improve the ability to target street medicine and other street-based services toward impactful interventions.

We also note that homeless individuals with multiple marginalized identities (e.g., racial/ethnic, gender, and sexual minorities) may be at heightened risk of poor health.^62^^,^^63^ More research is needed to understand how social inequalities by race, gender, and sexuality interact to shape health outcomes among people experiencing homelessness, especially unsheltered homelessness. This will require samples of adequate size to conduct stratified analysis and more rigorous methods to ensure representation.

A number of interventions offer the potential to improve the welfare of unsheltered individuals while also engaging clients on a pathway to housing. Street medicine programs can deliver much-needed services and engage clients with service systems, although challenges remain, including identification of high-impact service packages, coordinating care across fragmented service providers, and ensuring sustainability.^64^ Mobile phones are widely used among unsheltered individuals and can serve as a lifeline for emergency services and case worker outreach,^65^, ^66^, ^67^ but interventions are needed to improve access to connectivity and charging^68^ and to develop equity-sensitive digital service delivery models.^69^ Some interventions can increase the safety of unsheltered living arrangements, such as safe parking or camping areas.^70^ Finally, evidence from Japan suggests that cash transfers delivered through basic income programs or benefit enrollment initiatives may hasten the transition from the streets.^71^ Ultimately, however, any durable solution will require increased shelter inventory and a better understanding of the barriers to shelter entry.^61^

## CONCLUSIONS

Unsheltered populations experience high rates of chronic disease, serious mental illness, and substance abuse than sheltered populations. Unsheltered homelessness is strongly associated with chronic homelessness that exacerbates serious mental illness and substance use, which are often co-occurring. The rates of premature mortality are high relative to sheltered populations, and older adults are particularly vulnerable owing to accelerated aging while on the street. Despite having high unmet health needs, unsheltered populations have lower healthcare utilization and often lack health insurance. Results are consistently positive for most health conditions, but the evidence quality is mixed. Future research should include longitudinal studies that account for the timing and duration of homelessness; explore specific causal mechanisms of impact; and address intersectionality with race, ethnicity, sex, gender, sexual orientation, and other marginalized identities. Although further research is desperately needed, our results also suggest an urgent need to address the unique and severe challenges facing unsheltered populations and the need for intervention approaches that are sensitive to these challenges.

## CRediT authorship contribution statement

**Jessica Richards:** Conceptualization, Data curation, Formal analysis, Methodology, Resources, Software, Validation, Visualization, Writing – original draft, Writing – review & editing. **Randall Kuhn:** Conceptualization, Data curation, Formal analysis, Methodology, Resources, Supervision, Validation, Visualization, Writing – original draft, Writing – review & editing.

## Appendix Supplementary materials

Image, application 1

Image, application 2

## Footnotes

Supplementary material associated with this article can be found in the online version at doi:10.1016/j.focus.2022.100043.
