# Supplementary material for: Unsheltered Homelessness and Health: A Literature Review
Source: AJPM Focus. 2022 Oct 29;2(1):100043. doi: 10.1016/j.focus.2022.100043 (PMC10546518; doi:10.1016/j.focus.2022.100043)
Supplement: Supplementary file 2 [file mmc2.docx]

Appendix Table 1: Keywords used for PubMed literature review searches

| Keywords | Publications |
| --- | --- |
| 1. (((roofless*) OR (houseless*)) OR (homeless*)) OR (unhoused) | 12,995 |
| 1. (encampment*) | 76 |
| 1. (“sleep* rough”) OR (“rough sleep*”) | 143 |
| 1. ((“street homeless*”) OR (“street dwell*”)) OR (“street population*”) | 71 |
| 1. (nonshelter*) OR (unsheltered) | 130 |
| Total | 13,415 |
| De-duplicated Total | 13,407 |

Appendix Table 2: Criteria used for EndNote smart groups

| Endnote smart group | Criteria | Publications |
| --- | --- | --- |
| 1. Homelessness | Any field contains roofless* OR houseless* OR homeless OR unhoused | 12,984 |
| 1. Encampment | Any field contains encampment* | 76 |
| 1. Sleep rough | Any field word begins with sleep* AND rough* | 140 |
| 1. Street homeless | Any field contains street dwell* OR street-dwell OR street*dwell* OR street population* OR street-population* OR street*population* OR street, population* OR street homeless* OR street-homeless* | 68 |
| 1. Unsheltered | Any field contains unsheltered OR nonshelter* OR non-shelter* OR non-shelter-using | 130 |
| Total | (Homelessness) AND (Encampment) OR (Sleep rough) OR (Street homeless) OR (Unsheltered) | 174 |

Appendix Table 3: Number of publications excluded by reason

| Not in English (6) | (de Oliveira, 2007; Drogoul, 1996; Gomes et al., 2002; Pinzón-Rondón, Briceño-Ayala, Botero, Cabrera, & Rodríguez, 2006; Rosa Ada, Cavicchioli, & Brêtas, 2005; van Laere & Buster, 2001) |
| --- | --- |
| Published prior to 1990 (8) | (Atkinson, 1988; Brickner et al., 1986; Burt & Cohen, 1989; Cohen, Teresi, & Holmes, 1988a; Cohen, Teresi, & Holmes, 1988b; Gelberg & Linn, 1989; Hannappel, Calsyn, & Morse, 1989; Ramsden, Nyiri, Bridgewater, & el-Kabir, 1989) |
| Not original research (22) | (Brush, Gultekin, & Grim, 2016; Christensen, 2009; Courtwright, 1998; Crane & Warnes, 2001b; Depp, Vella, Orff, & Twamley, 2015; Donaldson, 2010; Farrell, Reissing, Evans, & Taylor, 2004; Fielding, 1998; Goode, Hoang, & Crocombe, 2018; Hurrell, 1994; Incze & Katz, 2018; Johnson, 1998; Kirkland-Kyhn, 2020; Latkin, 1998; Leshner, 1998; Lilja, Hamilton, & Larsson, 1998; Liu, Chai, & Watt, 2020; Neto et al., 2020; Peate, 2019; Raines & O'Connor, 2019; Ramanuj, 2019; Thomas, 2019) |
| Qualitative (29) | (Attenborough, 1998; Cunningham & Slade, 2019; Dickson-Gomez, Convey, Hilario, Corbett, & Weeks, 2007; Ellsworth, 2019; Fordham, 2015; Ghose, Boucicaut, King, Doyle, & Shubert, 2013; Haile, Umer, Ayano, Fejo, & Fanta, 2020; Håkanson & Öhlén, 2016; Hino et al., 2018; Hodgetts, Radley, Chamberlain, & Hodgetts, 2007; Howe, Buck, & Withers, 2009; Jagpal, Barnes, Lowrie, Banerjee, & Paudyal, 2019; Johnsen, Cloke, & May, 2005; Jordan, 2013; Kirkman, Keys, Bodzak, & Turner, 2010; Kryda & Compton, 2009; Lloyd, Page, McKeganey, & Russell, 2019; Myburgh, Moolla, & Poggenpoel, 2015; O'Carroll, Irving, O'Neill, & Flanagan, 2017; Parsell, Clarke, & Vorsina, 2020; Petrovich & Cronley, 2015; Salem & Ma-Pham, 2015; Shah, Koch, & Singh, 2019; Sumerlin, 1996b; Swart-Kruger & Richter, 1997; Swigart & Kolb, 2004; Ungpakorn & Rae, 2020; Wright, Oldham, & Jones, 2005; Wusinich, Bond, Nathanson, & Padgett, 2019) |
| Study design not applicable (17) | (Berry, 2007; Bourgois, 1998; Bourgois & Schonberg, 2007; Eyrich-Garg & Moss, 2017; Farrell & Reissing, 2004; Hopper, Shinn, Laska, Meisner, & Wanderling, 2008; O'Connell, Mattison, Judge, Allen, & Koh, 2005; Payne, 2002; Peterson, Baer, Wells, Ginzler, & Garrett, 2006; Pierangeli & Lenhart, 2018; Shern et al., 2000; Smith & Hall, 2018; Stergiopoulos et al., 2010b; Timms & Perry, 2016; Uddin et al., 2012; Uddin et al., 2009; van Hest & Story, 2009) |
| No health or health-related outcome (19) | (Early, 2005; Eyrich-Garg, 2010; K. M. Ferguson, 2007; Fischer, Shinn, Shrout, & Tsemberis, 2008; Gabrielian et al., 2016; Gallaher, Herrmann, Hunter, & Wilkins, 2020; Gory, Ritchey, & Fitzpatrick, 1991; Henderson et al., 2008; Hewett, 1998; E. E. Johnson, Borgia, Rose, & O'Toole, 2017; Larsen, Poortinga, & Hurdle, 2004; Lettner, Doan, & Miettinen, 2016; Loopstra et al., 2016; Mogk, Shmigol, Futrell, Stover, & Hagopian, 2020; Montgomery, Byrne, et al., 2016; Sumerlin, 1995, 1996a; Tsemberis, 1999; Tsemberis & Eisenberg, 2000) |
| Sheltered and unsheltered aggregated without comparison (45) | (Amato et al., 2019; Barrow & Medcalf, 2019; Black et al., 1991; Brett et al., 2014; Bymaster, Chung, Banke, Choi, & Laird, 2017; C et al., 2017; Chondraki, Madianos, Dragioti, & Papadimitriou, 2014; Coyle et al., 2015; G. M. Craig et al., 2007; T. K. Craig & Hodson, 2000; Crane & Warnes, 2001a; Darling, Palmer, & Kipke, 2005; Doughty, Stagnell, Shah, Vasey, & Gillard, 2018; Ensign, 2001; Ensign & Santelli, 1998; Fallaize, Seale, Mortin, Armstrong, & Lovegrove, 2017; Ferguson & Xie, 2012; Fichter & Quadflieg, 2001; Fisher, Turner, Pugh, & Taylor, 1994; Fisher et al., 2013; Gelberg, Gallagher, Andersen, & Koegel, 1997; Goldade et al., 2012; Green, Tucker, Golinelli, & Wenzel, 2013; Harris et al., 2020; Lam & Rosenheck, 1999; Lane et al., 2018; Lewer et al., 2019; Lewis & Ferguson, 2014; Marshall & Gath, 1992; Milaney, Kamran, & Williams, 2020; North & Smith, 1993; North, Smith, & Spitznagel, 1994; A. Nyamathi, Keenan, & Bayley, 1998; O'Toole et al., 1999; O'Toole, Johnson, Borgia, & Rose, 2015; Patanwala et al., 2018; Pluck, Barajas, Hernandez-Rodriguez, & Martínez, 2020; Poulin, Maguire, Metraux, & Culhane, 2010; Reyes et al., 2005; Salomonsen-Sautel et al., 2008; Santa Maria, Padhye, Yang, Gallardo, & Businelle, 2018; Shaw, Dorling, & Brimblecombe, 1999; Stratigos et al., 1999; Thompson, 2004; Topp et al., 2013) |
| Child/youth sample (8) | (De Rosa et al., 1999a; Ensign & Santelli, 1997; Forde, Baron, Scher, & Stein, 2012; Ginzler, Garrett, Baer, & Peterson, 2007; Greene et al., 1997; Moore et al., 2019; Ray et al., 1999; Rhoades, Winetrobe, & Rice, 2014) |
| Distinction between sheltered and unsheltered unclear (5) | (Hofmeister et al., 2019; Hwang, 2002; Levitt et al., 2012; Stafford & Wood, 2017; Yun et al., 2003) |
| Did not meet methodological criteria (4) | (Ayano et al., 2017; Gambatese et al., 2013; Harney et al., 2019; Llerena, Gabrielian, & Green, 2018) |

**Appendix Table 4: Inclusion criteria and quality scoring**

|  | Points |
| --- | --- |
| Tier 1 – Do they use multivariate statistics to adjust the health difference in sheltered/unsheltered to account for demographic factors?  Tier 2 – Does the study provide demographic background data that would make it possible to conduct adjustments? | Yes (1)  No (0) |
| Tier 1 and 2 – Sampling:   - Does the paper describe a sampling strategy or was it based strictly on convenience? - Does the sample consist of volunteers or people who were selected for interview? Specifically, is a sampling strategy given and applied? - Was any attempt made to have the sample be representative of the broader sheltered and unsheltered homeless population? Or was recruitment site selection strictly based on convenience? If selection took place at multiple sites, was thought given or were analyses conducted to assert the appropriateness of this approach?   Tier 1   - Did the sampling mechanisms or selection probability differ for sheltered and unsheltered individuals without any effort to adjust for this bias? | Probability sample (1)  Convenience sample not selected on health (1)  Convenience sample selected on health (0) |
| Tier 1 and 2 – Measurement:   - Were the health measures validated and collected with proper training and quality assurance? | Yes (1)  No (0) |
